# Detecting Multiple Myeloma Infiltration of the Bone Marrow on CT Scans in Patients with Osteopenia: Feasibility of Radiomics Analysis

**DOI:** 10.3390/diagnostics12040923

**Published:** 2022-04-07

**Authors:** Hyerim Park, So-Yeon Lee, Jooyeon Lee, Juyoung Pak, Koeun Lee, Seung-Eun Lee, Joon-Yong Jung

**Affiliations:** 1 Department of Radiology, Seoul St. Mary’s Hospital, College of Medicine, The Catholic University of Korea, Seoul 06591, Korea; hprad@schmc.ac.kr (H.P.); susan505@hanyang.ac.kr (J.L.); fanyi02@catholic.ac.kr (K.L.); sseung329@catholic.ac.kr (S.-E.L.); messengr@catholic.ac.kr (J.-Y.J.); 2 Department of Radiology, Soonchunhyang University Cheoan Hospital, Cheonan 31151, Korea; 132633@schmc.ac.kr; 3 Department of Applied Statistics, Hanyang University, Seoul 04763, Korea

**Keywords:** multiple myeloma, computed tomography, radiomics, texture analysis, machine learning

## Abstract

It is difficult to detect multiple myeloma (MM) infiltration of the bone marrow on computed tomography (CT) scans of patients with osteopenia. Our aim is to determine the feasibility of using radiomics analysis to detect MM infiltration of the bone marrow on CT scans of patients with osteopenia. The contrast-enhanced thoracic CT scans of 104 patients with MM and 104 age- and sex-matched controls were retrospectively evaluated. All individuals had decreased bone density on radiography. The study group was divided into development (n = 160) and temporal validation sets (n = 48). The radiomics model was developed using 805 texture features extracted from the bone marrow for a development set, using a Random Forest algorithm. The developed models were applied to evaluate a temporal validation set. For comparison, three radiologists evaluated the CTs for the possibility of MM infiltration in the bone marrow. The diagnostic performances were assessed and compared using an area under the receiver operating characteristic curve (AUC) analysis. The AUC of the radiomics model was not significantly different from those of the radiologists (*p* = 0.056–0.821). The radiomics analysis results showed potential for detecting MM infiltration in the bone marrow on CT scans of patients with osteopenia.

## 1. Introduction

Multiple myeloma (MM) is a hematologic malignancy characterized by abnormal production of the monoclonal immunoglobulin M component of plasma cells in the bone marrow [1]. Globally, multiple myeloma contributes to 1% of all cancer deaths. MM is most commonly identified in patients who are 65–70 years of age, and is more prevalent in men than in women [2]. MM is a spectrum of diseases that encompasses a monoclonal gammopathy of undetermined significance, smoldering MM, and classic MM [3]. The International Myeloma Working Group defined MM as the presence of at least 10% clonal bone marrow plasma cells, or biopsy-proven bony or extramedullary plasmacytoma, and any later myeloma-defining events. Myeloma-defining events include end-organ damage, attributable to underlying plasma cell proliferative disorders (hypercalcemia, renal insufficiency anemia, and bone lesions) [4].

After metastasis, MM is the most frequently diagnosed type of skeletal cancer [5]. Bone involvement is one of the most prominent features of MM, and up to 80% of patients with this condition develop bone disease on radiography as part of a skeletal survey [6]. Parts of the axial skeleton, such as vertebral bodies (49%), skull (35%), and ribs (33%), are frequently affected [7]. Multiple modalities, including radiography, computed tomography (CT), magnetic resonance imaging (MRI), and positron emission tomography/CT, can be used to evaluate bone involvement [4,8]. 

In recent years, low-dose, whole-body CT has been recommended to diagnose MM bone disease, rather than radiography [8]. Whole-body, low-dose CT has demonstrated a superior diagnostic performance compared to radiography [9,10,11,12]. Its ability to detect osteolytic lesions smaller than 5 mm, as well as lesions 5 mm or greater, is satisfactory [13]. However, MM frequently occurs in older people with generally reduced bone density. Thus, osteolytic lesions are sometimes hard to distinguish from osteoporotic bones, and osteoporosis itself mimics osteolytic lesions. Regarding non-osteolytic lesions, CT is typically unable to detect hyperdense focal lesions within the vertebral bodies, due to the presence of trabeculations [14,15]. 

Radiomics is an emerging technique for the quantitative evaluation of medical imaging. A large number of imaging features, which are termed radiomics features, can be extracted using specific algorithms. This quantitative analysis has the potential to detect additional characteristics that the traditional qualitative analysis fails to identify [16]. Recent studies on CT radiomics have shown the possibility to detect bone pathology, which was not easily identified using human visual assessment alone [17,18,19]. A study by Muehlematter et al. [17], on 60 stable and 60 unstable vertebrae of 58 patients, found that radiomics with machine learning showed an area under the receiver operating characteristic curves (AUCs) of 0.88–0.97 for identifying patients at risk of insufficiency fractures. In a study by Tagliafico et al. [18], regarding differentiation between 22 diffuse and 39 focal lesions in 70 patients with MM, the AUC of radiomics was 0.68–0.79, while the AUC of the radiologists was 0.64. Preliminary reports by Kakigi and colleagues [19] found that a texture analysis of the L3 vertebral body on contrast-enhanced spine CT revealed differences among patients with normal bone density (n = 15), with osteoporosis (n = 13), and with MM (n = 10) (*p* < 0.05). 

We hypothesized that the detection of multiple myeloma infiltration in bone marrow on CT can be achieved using a radiomics model. The aim of this preliminary and retrospective case–control study was to develop and evaluate the diagnostic performance of a CT radiomics model for the detection of MM infiltration in the bone marrow on CT scans in patients with osteopenia.

## 2. Materials and Methods

### 2.1. Patient Population

**Figure 1:**
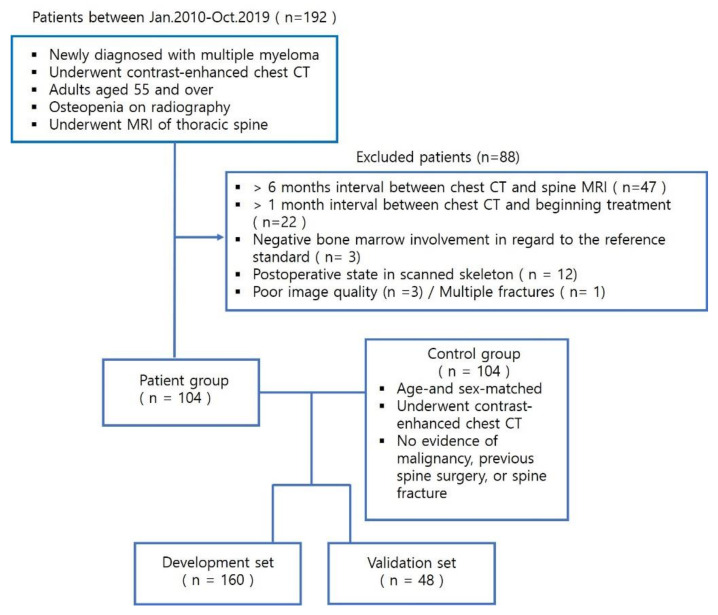
A flow chart of the participant selection process.

The Institutional Review Board waived the requirement for informed consent for this retrospective study. The inclusion criteria were as follows: consecutive patients who were newly diagnosed with MM at our institution between January 2010 and October 2019; patients who underwent contrast-enhanced chest CT; adults aged 55 and over; patients with osteopenia on radiography; and patients who underwent MRI of the thoracic spine. In all, 192 patients met the inclusion criteria. The exclusion criteria were (1) >6-month interval between chest CT and spine MRI, (2) >1-month interval between chest CT and beginning treatment, (3) negative bone marrow involvement in regard to the reference standard, (4) postoperative state in scanned skeleton, and (5) other reasons, including poor image quality and multiple fractures. We excluded 88 patients, and the remaining 104 patients were enrolled in this study cohort as the patient group (Figure 1). The images of 104 patients who underwent contrast-enhanced chest CT at our institution, without evidence of malignancy, were matched by age and sex for the control group. All individuals clearly had osteopenia on radiological examination. The total study cohort was divided into a development set and a validation set, according to the examination time. The patients who underwent CT in 2018 and 2019 were assigned to the validation group (n = 48), and all others were assigned to the development group (n = 160).

### 2.2. CT Data Acquisition

Chest CT was performed using various scanners: 64-detector rows (Somatom definition, Siemens Medical System, Erlangen, Germany; Discovery CT 750 HD, GE Healthcare, Waukesha, WI, USA) or 128-detector rows (Definition AS+, Siemens Medical System, Erlangen, Germany) for one breath hold. The scans were obtained from the level of the lung apices to the adrenal glands. The scanning parameters included a 130–293 mAs tube current, a 100–140 kV tube voltage, and a variable scan thickness of 2.5–5 mm section thickness, without a specific scan interval. All CT examinations were performed with intravenous contrast material (Iopromide (Ultravist 300), Bayer Schering Pharma AG, Berlin, Germany; ioversol (Optiray 320 mg/mL), Mallinckrodt Pharmaceuticals, Dublin, Ireland).

### 2.3. Lesion Segmentation and Radiomics Feature Extraction

**Figure 2:**
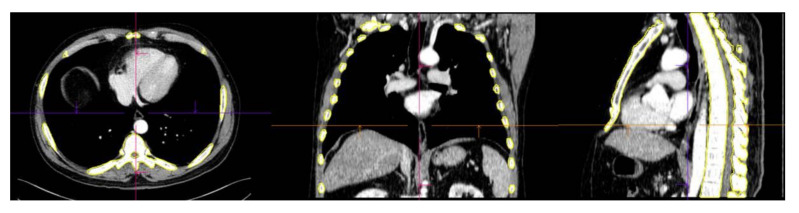
Segmentation of the axial skeleton.

Image segmentation and feature extraction were performed on contrast-enhanced chest CT scans using a prototype software program (syngo.via Frontier, version 1.2.2, Siemens Healthineers, Munich, Germany). Segmentation of the entire skeleton (including the spine, rib cage, sternum, clavicles, and scapulae) was performed using an automatic lesion segmentation tool within the software (Figure 2). The radiomics features of the segmented volume of interest (VOI) were extracted using the publicly available PyRadiomics library (PyRadiomics library, version 3.0.1. Available online: https://githumb.com/Radiomics/pyradiomics (accessed on 25 Mar 2021)). To correct the variability that resulted from parameters related to voxel size, the segmented volume of interest (VOI) was resampled to isometric voxels of 1 × 1 × 1 mm^3^ [20]. The radiomics features of the resampled images were extracted using the publicly available PyRadiomics library (R statistical software, version 3.5.2, Vienna, Austria. Available online: https://cran.r-project.org/bin/windows/base/old/3.5.2/ (accessed on 12 May 2021)). Radiomics features were subdivided into the following classes: first-order features (18 features), volume and shape features (14 features), texture-based features (68 features), wavelet-filtered features (688 features), and Laplacian of Gaussian-filtered features (430 features). One radiologic technologist (K.E.L.) performed image segmentation and feature extraction under the supervision of a radiologist (S.Y.L.).

An automatic lesion segmentation tool for the skeleton in the software was used to calculate the VOI for the spine, rib cage, sternum, clavicles, and scapulae.

### 2.4. Dimensionality Reduction and Radiomics Feature Selection

**Figure 3:**
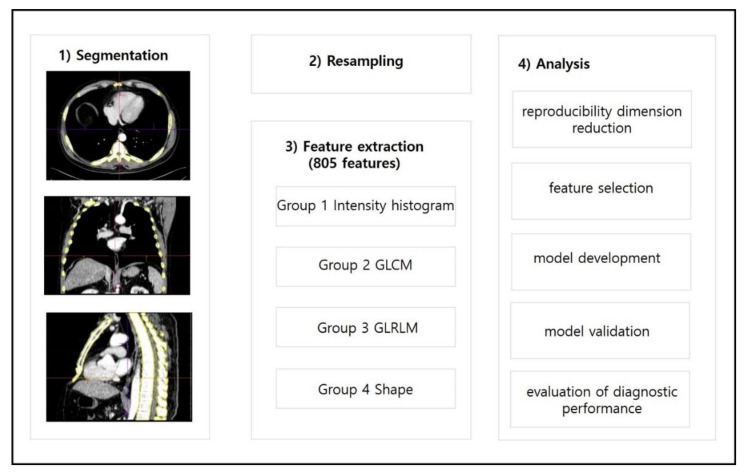
A methodological flow chart of this study (GLCM, Gray Level Co-occurrence Matrix; GLRLM, Gray Level Run Length Matrix).

To determine the reproducibility and avoid dimensionality, two steps were adopted to select the features (Figure 3). First, one radiologist (H.P.) independently carried out auto-segmentation of the CT images using a prototype software program (syngo.via Frontier, version 1.2.2, Siemens Healthineers, Munich, Germany) and feature extraction for randomly selected samples (n = 87) to evaluate the reproducibility of segmentation and quantification [21]. The intraclass correlation coefficient (ICC), based on a single-measurement, absolute-agreement, two-way random-effects model, was used to evaluate the inter-observer agreement [22]. An ICC score >0.95 was considered to indicate satisfactory agreement. The features that were extracted with an ICC >0.95 were considered reproducible parameters and were selected for radiomics model construction [23]. Second, a binomial elastic net was used to shrink the features that were strongly associated with the outcome. All features that were correlated with each other were removed using the elastic net. The number of features to be selected was determined by fine tuning the optimal alpha and lambda values to achieve a good fit and to avoid overfitting, and this was carried out using 10-fold cross-validation. Calculations of the ICC and binomial elastic net were performed with commercially available software (R statistical software, version 3.5.2, Vienna, Austria. Available online: https://cran.r-project.org/bin/windows/base/old/3.5.2/ (accessed on 12 May 2021)).

### 2.5. Radiomics Model Development

A Random Forest (RF) machine learning algorithm was used to build the classifier models with commercially available software (R statistical software, version 3.5.2, Vienna, Austria. Available online: https://cran.r-project.org/bin/windows/base/old/3.5.2/ (accessed on 12 May 2021)). The generalization capacity of the model was evaluated using cross-validation with at least ten folds of multiple experiments. The feature importance scores were obtained for all cross-validation experiments.

### 2.6. Radiomics Model Validation

Validation of the radiomics model was performed using a temporal validation set that consisted of patients both with (n = 24) and without (n = 24) MM. To evaluate the diagnostic performance of the model, the CT images were independently reviewed by three radiologists who were blinded to the patients’ diagnoses: two board-certified radiologists with 10 years (S.Y.L., R1) and 1 year (H.P., R2) of experience in musculoskeletal imaging, respectively, and one radiologist (J.P., R3) with 1 year of training. The first of two steps in this process was to directly compare the diagnostic performance of the radiomics model with those of the radiologists. The readers examined each patient and rated the possibility of MM involvement in the bone marrow using a 5-point scale (0–4: definitely negative, probably negative, possibly positive, probably positive, and definitively positive, respectively) [11]. These readers reviewed the images on the bone window setting (window level: 1800 HU; window width: 400 HU). However, the readers were able to change the window width and level on the picture archiving and communication system if needed. To avoid recall bias, three readers selected and segmented the lesions, and then evaluated the CT images three months later. 

### 2.7. Evaluation of the Usefulness of Adding a Radiomics Model to Conventional Reading

The second step was to determine whether or not the diagnostic performances improved when radiologists used additional diagnostic information from radiomics models. After rating the possibility of MM in the first step, readers were provided with information on whether the radiomics model assessed the patient as MM or normal. Then, they re-rated the possibility of MM after correlating the classification results obtained from the radiomics model. The first and second steps of CT review were conducted sequentially for each patient. The readers who were blinded to the diagnosis independently rated the possibility of MM involvement in the bone marrow using a 5-point scale [11].

### 2.8. Statistical Analysis

MR images were used as the standard of reference. The sensitivity, specificity, accuracy, and AUC data for the developed model were determined. Three radiologists examined the validation set. The sensitivity, specificity, and accuracy of the radiomics model and of the three radiologists were compared using the McNemar test. The AUCs were compared between the models using the DeLong test, using commercially available software (MedCalc Statistical Software, version 19.2.1, Medcalc Software Ltd., Ostend, Belgium).

## 3. Results

### 3.1. Patient Characteristics

The mean age of the patients in the development set was 63.8 years (range: 56–79 years), while that of the validation set was 63.5 years (range: 57–74 years). There were 81 women and 79 men in the development set, and 27 women and 21 men in the validation set. The control groups of both the development and validation sets had the same mean age and sex proportions. There were various clinical indications for undergoing CT examination in the control group, as follows: chest discomfort (n = 21), respiratory symptoms (n = 61), fever (n = 5), screening (n = 7), trauma (n = 2), and miscellaneous (connective tissue disease = 3, lipoma = 1, cardiac symptoms = 2, and wound infection = 2).

### 3.2. Radiomics Feature Selection and Model Development

In total, 1218 radiomics features were extracted from the skeleton using semi-automatic segmentation. Among them, 805 features were selected as reproducible features (Supplementary Materials Appendix S1). Elastic net selected 48 radiomics features using optimal parameters (lambda = 0.018664 and alpha = 0.729, respectively). The RF classifier was developed based on the selected features. The feature importance was calculated based on the mean decrease in node impurities, measured by the Gini Index, from the developed model. An RF-based model was developed. The top 10 features, according to importance, are presented in Table 1. The sensitivity, specificity, accuracy, and AUC of the radiomics model were 0.76 (95% confidence interval [CI]: 0.65–0.85), 0.78 (95% CI: 0.67–0.86), 0.77 (95% CI: 0.70–0.83), and 0.858 (95% CI: 0.801–0.916), respectively. Confidence intervals from 2000 bootstrapped samplings were obtained to determine the accuracy and AUC, while exact binomial confidence limits were calculated to determine the sensitivity and specificity.

### 3.3. Diagnostic Performance of the Radiomics Model

**Figure 4:**
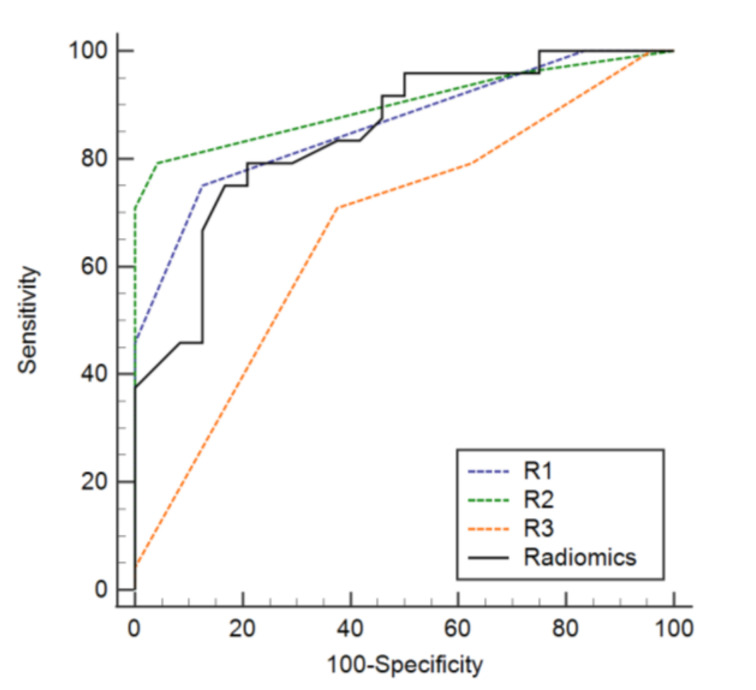
Diagnostic performance of the radiomics model and the three readers.

In the validation set, the sensitivity, specificity, accuracy, and AUC of the radiomics model were 0.75 (0.530.90), 0.83 (0.63–0.95), 0.79 (0.65–0.90), and 0.846 (0.737–0.955), respectively (Table 2). The sensitivity, specificity, accuracy, and AUC of R1 were 0.75 (0.53–0.90), 0.88 (0.68–0.97), 0.81 (0.70–0.93), and 0.862 (0.770–0.954), respectively. For R2, the sensitivity, specificity, accuracy, and AUC were 0.79 (0.58–0.93), 0.96 (0.79–1.00), 0.88 (0.78–0.97), and 0.900 (0.811–0.989), respectively. The sensitivity, specificity, accuracy, and AUC of R3 were 0.79 (0.58–0.93), 0.38 (0.19–0.60), 0.58 (0.44–0.73), and 0.668 (0.509–0.790), respectively (Table 3). The specificity of the radiomics model was superior to that of R3 (*p* = 0.019), whereas there was no difference between the results of R1 and R2 (*p* ≥ 0.375). There was also no significant difference among the sensitivities and accuracies of the three radiologists and the radiomics model. The AUC scores of the radiomics model were not significantly different amongst radiologists (*p* ≥ 0.056) (Figure 4).

### 3.4. Added Value of a Radiomics Model to Conventional Readings

**Figure 5:**
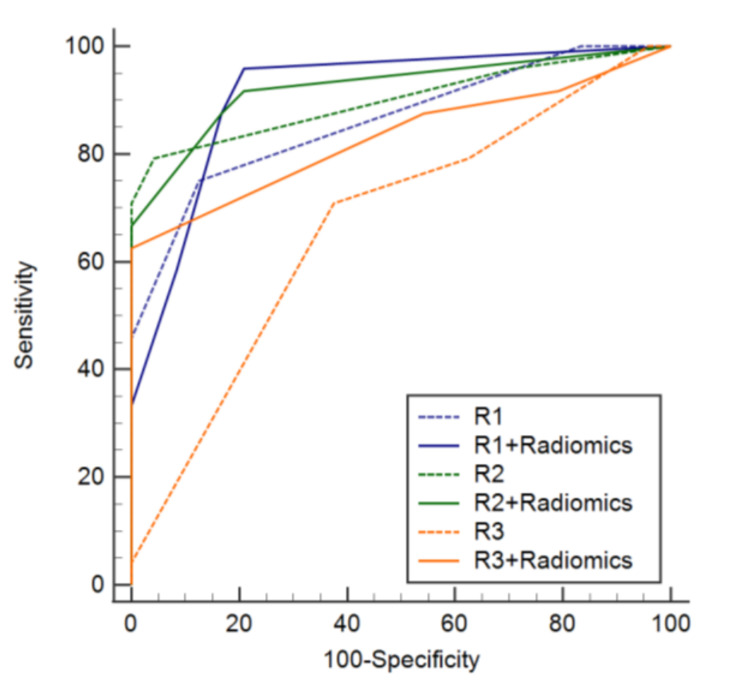
Diagnostic performance of the three readers with and without overall correlation with the results from the radiomics model.

The sensitivity, specificity, and accuracy of the diagnosis of MM in a second analysis, with a radiomics model, were not significantly different from the first review, without a radiomics model, for all the radiologists (Table 4; *p* ≥ 0.375). The AUC of the second analysis of R3, along with a radiomics model, was superior to that of the first review, without the radiomics model. The AUCs of the first and second analyses of R1 and R2 were not different (*p* ≥ 0.221) (Figure 5).

## 4. Discussion

The radiomics analysis showed potential for detecting MM infiltration in the bone marrow on CT scans of patients with osteopenia in this study. The diagnostic performance of the radiomics-based machine learning model was as high as that of an experienced radiologist, and the specificity of the radiomics model was higher than that of an inexperienced radiologist. The radiomics model might be useful for assisting inexperienced radiologists or physicians in detecting MM infiltration in elderly patients with osteopenia. 

Our study also demonstrated that the addition of a radiomics-based analysis increased the sensitivity of the inexperienced radiologist for detecting MM; AUC: 0.668–0.830 for R3. There are several explanations for these findings. First, the radiomics study detected more non-osteolytic lesions than the visual assessment alone [14]. According to previous research into dual-energy CT in 34 patients with MM, a virtual non-calcium technique showed higher sensitivity compared to standard CT images (91.3% vs. 69.6%), due to the detection of non-osteolytic lesions. Second, the ability to discriminate osteolytic lesions from osteoporosis was higher in the radiomics analysis compared to the qualitative analysis [17,19]. According to previous studies that have used a radiomics analysis, osteoporosis could be differentiated from normal bone and MM infiltration [17,19].

A previous study by Mahnken et al. revealed that staging by MR imaging and radiography in some MM patients might be undervalued. Up to 20% of patients with biopsy-proven bone marrow infiltration showed normal findings on MRI [24]. Whole-body multi-detector computed tomography (MDCT) was superior to whole-body MRI for detecting residual osteolytic abnormalities in MM patients, as well as for exploring bones, such as ribs, clavicles, and skull [14,24,25,26,27]. Homman et al. reported that the visual assessment of rib involvement in MM depended on the thickness of the transverse CT images, with a sensitivity of 79.7%–88.1%, a specificity of 94.7–93.0%, and an accuracy of 87.1–90.5% [28]. It showed similar diagnostic accuracy to our study for detecting MM involvement. In addition, among other imaging modalities, CT is beneficial in comparison, across multiple institutions and vendors using phantom [16]. Therefore, CT-based radiomics analysis could potentially be a good predictor for the detection of MM.

Demineralization is a common feature of MM. Dual-energy X-ray absorptiometry (DXA) is the most commonly used modality for the evaluation of osteoporosis [29]. Therefore, DXA is used as a marker of the treatment response of MM [30]. It is not well known if DXA differentiates demineralization related with MM and that of senile osteoporosis. Except for quantitative computed tomography (QCT), CT is not intended to evaluate osteoporosis. However, osteoporosis is common in the elderly, and it is problematic to mask or mimic MM on CT performed for other purposes. In this case, CT-based radiomics analysis provides additional information, without increased medical expense.

There are several limitations to our study. We did not evaluate the diagnostic performance of patients with MM with bone marrow infiltration and patients with MM without bone marrow infiltration. This topic seems be more clinically beneficial than that of our study. However, the differentiation of patients with MM with bone marrow infiltration and patients who only have osteopenia without MM is also useful for radiologists who report the CT findings of elderly patients in their daily practice. A radiomics model allows radiologists to increase their confidence when reporting the possibility of bone marrow infiltration in elderly patients. This was a retrospective study, and it might have involved selection bias. Some patients who had not undergone CT or MRI were excluded. As a result, the MRI findings used as the reference standard might not reflect the exact status of MM in a similar population. Automated segmentation was used to enhance the reproducibility; the VOI included the intervertebral discs, because the software we used failed to separate soft tissues from bony structures. It is possible that this value could have affected the overall results. We conducted a per-person analysis, rather than a per-lesion analysis. Therefore, we could not perform subgroup analyses, such as osteolytic vs. non-osteolytic lesions or rib lesions vs. vertebral lesions. The diagnostic performance of the radiomics model is likely to be affected by these factors. Some of the cervical vertebrae and lumbar vertebrae were also included, and the extent of inclusion likely varied from patient to patient [30]. 

As this study focused on the feasibility of the radiomics model, further research on the generalization of this model is needed for clinical application. The radiomics features were evaluated using a single software program. External validation with data from other institutes or other software was not performed, and this might have limited our findings. The CT parameters were diverse in this study, which affected the radiomics features. However, previous studies have shown that the tube current and tube voltage of CT have little effect on the radiomics features [31,32]. Radiomics features can vary depending on the CT scanner, so it is difficult to predict whether this model will be generalizable to other CT scanners. Additional training with bones other than the pelvic bone is needed for generalization, as an analysis of thoracic bone structures only is not enough to evaluate the tumor burden of MM.

The radiomics analysis demonstrated an equivalent diagnostic performance to the experienced radiologists’ findings. There is evidence that quantitative analysis using radiomics analysis can improve inexperienced radiologists’ evaluations of MM involvement on CT scans in elderly patients with osteopenia. Although these preliminary results must be interpreted with caution, until validated by an independent data set, our study has demonstrated the feasibility and usefulness of the radiomics analysis of CT images for the detection of MM. In conclusion, a bone texture analysis could be useful as one of the imaging markers of MM.

## Figures and Tables

**Table 1:** Top ten Random Forest feature importance for diagnosis of bone marrow involvement of multiple myeloma.

Radiomics Features	Importance
wavelet_HLL_glcm_Imc2	6.284976
wavelet_LLL_glcm_Imc2	3.911729
wavelet_HHH_glszm_SmallArea Emphasis	3.730171
wavelet_LLL_gldm_Dependence Entropy	3.620143
wavelet_LHL_glcm_Imc1	3.452086
wavelet_HLH_glcm_Correlation	2.614805
wavelet_HHL_glcm_Idmn	2.315703
wavelet_LHH_glszm_SmallAreaLowGrayLevelEmphasis	1.965419
wavelet_HLH_glcm_MCC	1.776747
wavelet_LHH_glrlm_LongRunLowGrayLevelEmphasis	1.77367

**Table 2:** Diagnostic performance of radiomics model.

	Sensitivity	Specificity	Accuracy	AUC
Development set	0.76 (0.65–0.85)	0.78 (0.67–0.86)	0.77 (0.70–0.83)	0.858 (0.801–0.916)
Validation set	0.75 (0.53–0.90)	0.83 (0.63–0.95)	0.79 (0.65–0.90)	0.846 (0.737–0.955)

Note—AUC, area under the receiver operating characteristic curve. Numbers within parentheses are 95% confidence intervals.

**Table 3:** Comparison of diagnostic performance of radiomics model and radiologists.

Diagnostic Performance	Sensitivity	Specificity	Accuracy	AUC
Radiomics model (A)	75% (18/24)	83% (20/24)	79% (38/48)	0.846 (0.737–0.955)
Readers(B)				
R1	75% (18/24)	88% (21/24)	81% (39/48)	0.862 (0.770–0.954)
R2	79% (19/24)	96% (23/24)	88% (42/48)	0.900 (0.811–0.989)
R3	79% (19/24)	38% (9/24)	58% (28/48)	0.668 (0.526–0.810)
Comparison of A and B				
R1	1.000	1.000	1.000	0.821
R2	1.000	0.375	0.424	0.451
R3	1.000	0.019 *	0.076	0.056

Note—sensitivity, specificity, and accuracy were compared using McNemar test; AUCs were compared using DeLong’s test; *, *p* value < 0.05.

**Table 4:** Diagnostic performance of radiologists after correlation with results from radiomics model.

Diagnostic Performance	Sensitivity	Specificity	Accuracy	AUC
Readers R1	88% (21/24)	83% (20/24)	85% (41/48)	0.912 (0.832–0.993)
R2	88% (21/24)	83% (20/24)	85% (41/48)	0.924 (0.851–0.998)
R3	88% (21/24)	46% (11/24)	67% (32/48)	0.83 (0.712–0.947)

## Data Availability

The datasets generated during and/or analyzed during the current study are available from the corresponding author on reasonable request.

## Supplementary Materials

The following supporting information can be downloaded at: https://www.mdpi.com/article/10.3390/diagnostics12040923/s1, Appendix S1: Features selected as reproducible features.
